# Current practice and awareness of pediatric off-label drug use in Shanghai, China -a questionnaire-based study

**DOI:** 10.1186/s12887-019-1664-7

**Published:** 2019-08-13

**Authors:** Mei Mei, Hong Xu, Libo Wang, Guoying Huang, Yonghao Gui, Xiaobo Zhang

**Affiliations:** 1 Department of Respirology, Children’s Hospital of Fudan University, 399 Wan Yuan Road, Shanghai, 201102 China; 2 Department of Nephrology, Children’s Hospital of Fudan University, Shanghai, China; 3 Cardiovascular Center, Children’s Hospital of Fudan University, Shanghai, China

**Keywords:** Awareness, Children, Off-label drug use, Practice, Questionnaire

## Abstract

**Background:**

Off-label drug use is widespread in pediatric drug treatment, and the implementation of guidelines on this topic remains challenging. The objective of this study was to evaluate current practice and awareness of healthcare professionals towards pediatric off-label drug use, as well as the barriers to guideline implementation among pediatric healthcare professionals in Shanghai, China.

**Methods:**

A validated questionnaire was issued to representatives of pediatricians, pharmacists, nurses and administrators from hospitals with pediatric qualification in Shanghai.

**Results:**

A total of 679 completed questionnaires from 69 hospitals were included in the analysis. Nearly half (47.9%) of the pediatricians acknowledged that they had prescribed off-label drugs. Most (88.4%) of the pharmacists acknowledged that they had dispensed off-label medicines. The main reason for off-label prescribing was the lack of pediatric dosage information. The most common category of off-label prescribing in children was dosage. Nearly half (42.0%) of the participating hospitals had developed internal protocols for off-label drug use. However, approximately half of the respondents reported that they did not adhere to the guidance and that it had barriers to implementation. Most respondents (84.5%) declared that they were familiar with the term “off-label drug use”. However, the awareness rate of the *Chinese Expert Consensus of Pediatric Off-Label Drug Use* was low (45.7%). More than half (55.4%) of the respondents declared that they did not adhere to the process proposed in the consensus and that barriers existed for its utilization.

**Conclusions:**

Pediatric off-label drug use is widespread in Shanghai, China, and barriers exist to the implementation of the guideline. A legally recognized national guideline with a broad scope of application for off-label drug use is urgently needed; at the same time, more education and training on off-label drug use should be provided to targeted healthcare professionals.

**Electronic supplementary material:**

The online version of this article (10.1186/s12887-019-1664-7) contains supplementary material, which is available to authorized users.

## Background

Off-label drug use is defined as “drugs prescribed and used outside their licensed indications with respect to dosage, age, indication, or route” [1]. Due to the lack of drugs specifically designed and marketed for children, off-label drug use is very common in pediatric drug treatment. Data from specialized children’s hospitals in China revealed that off-label drug use rates ranged from 46.9–95% in pediatric wards and 53–82.7% in pediatric outpatient departments, respectively [2, 3].

Although the existence of off-label drug use has its rationality, it may cause several problems. First, off-label prescribing can jeopardize patient safety in certain clinical scenarios where a positive benefit-risk ratio is not fully established. This is mainly due to the fact that off-label drug use is not systematically appraised by regulators, guideline formulators or even healthcare policymakers. Second, off-label use raises issues of liability in the case of adverse events which makes physicians vulnerable to potential legal sanctions. Moreover, drugs used in an off-label manner are usually not reimbursed and would ultimately increase costs to patients and society [4–6]. Due to its high prevalence and regulatory challenge in pediatric medical practice, off-label drug use has become a worldwide problem [6–9].

The regulations pertaining to the practice of off-label drug use have not been harmonized across the world. In some developed countries, such as United States, France and Britain, national legislations, regulations or guidelines concerning off-label drug use have been established, and rational off-label drug use is allowed in these countries [10–12]. In India, off-label prescribing is illegal according to the Amendments to the Indian Medical Council Act 2 [13]. However, there is no clear description of off-label prescribing according to Chinese laws [14]. In the past few years, great efforts have been made to improve this situation. In 2016, the *Chinese Expert Consensus of Pediatric Off-Label Drug Use* was published in the Chinese Journal of Pediatrics, which was written by the Chinese Pediatric Society [15]. The expert consensus was intended to increase awareness of off-label drug use among pediatric healthcare professionals and to provide a practical and explicit approach to off-label prescribing that would ultimately result in improved drug use in children. The expert consensus recommended the following process for off-label drug use: (1) Requesting access to off-label drug use based on supportive evidence; (2) Being assessed by the expert group of off-label drug use; (3) Being approved by the ethics committee and/or the pharmacy administration committee; (4) Obtaining informed consent; (5) Monitoring the adverse drug reactions (ADRs); and (6) Establishing a database of the off-label drugs and updating it regularly. Though the consensus had little legal force, it was a positive exploration of standardized pediatric off-label drug use.

To date, several studies have been carried out to assess the awareness and experiences of different healthcare professionals towards pediatric off-label drug use in Western countries [16–21], but none of them assessed the barriers to guideline implementation in regards to this topic. Furthermore, little is known about the situation in China, a developing country with a different medical system. To evaluate current practices and awareness of pediatric off-label drug use as well as barriers to the implementation of the current expert consensus in Shanghai, China, we conducted a cross-sectional questionnaire study.

## Methods

### Study design, setting and participants

This cross-sectional study was conducted from July 5 to August 5 in 2017 in partnership with Shanghai Pediatric Clinical Quality Control Center (SPQCC), an organization focused on improving pediatric medical security and quality led by the Shanghai Municipal Commission of Health and Family Planning. The center is responsible for providing clinical guidance to hospitals with pediatric qualification in Shanghai, including four specialized children’s hospitals (all tertiary hospitals) and 65 general hospitals (42 secondary hospitals and 23 tertiary hospitals). Eligible participants were registered healthcare professionals in the 69 member hospitals of SPQCC and were involved in off-label drug use. In each hospital, we divided the population into strata based on four important professional characteristics (i.e., pediatricians, pharmacists, nurses and administrators). Simple random samples were selected from each stratum using random numbers based on employee number. The participants were allowed two weeks to return the completed questionnaire. The study completeness and data quality were ensured by two trained researchers who checked the integrity and audited the data of the completed questionnaires. The study was reviewed and approved by the ethics committee of the Children’s Hospital of Fudan University (2017–263). A letter from SPQCC was sent to participants to inform them about the significance and use of the questionnaire, and informed consent was deemed to be given after completion of the questionnaire.

### Questionnaire

An online anonymous questionnaire comprising 50 questions was developed on the basis of previous similar surveys [16–18] and in consideration of the expert consensus written by the Chinese Pediatric Society in 2016. The content validity of the questionnaire was evaluated by a multidisciplinary group discussion of experts in pediatrics, pharmacology, nursing and epidemiology. The questionnaire focused primarily on the off-label drug use in children and consisted of three sections. The first section focused on participants’ general information (six items). The second section focused on the off-label prescribing, dispensing and administering experiences of the participants and the management of off-label drug use in the participants’ units (11 items). The third section focused on the awareness of healthcare professionals regarding off-label drug use and the *Chinese Expert Consensus of Pediatric Off-Label Drug Use* (33 items). The definition of off-label drug use was provided as an integral part of the questionnaire to guide respondents who were not familiar with the terminology (see Additional file 1). The questionnaire was pilot tested with 67 healthcare professionals including pediatricians, pharmacists, nurses and administrators. The reliability of the questionnaire was confirmed by its Cronbach’s alpha value of 0.87.

### Statistical analysis

We estimated the sample size using EpiTools Epidemiological Calculators (http://epitools.ausvet.com.au). A minimum sample size of 385 was required taking into account the expected off-label awareness rate of 50% with the 95% confidence interval and the need for 5% precision. Considering the stratified sampling with four important characteristics within 69 hospitals, 828 samples were needed to ensure at least 3 samples within each stratum, then, a total of 828 participants were required for this study. Data were downloaded as Microsoft Excel format and subsequently analyzed in SPSS version 19.0. Questionnaires with missing data (if any question was unanswered) or contradictory answers (if any answers were at odds with each other or with apparent inconsistency with the truth) were excluded. Descriptive analysis has been used for analyzing various categorical variables. Chi-square test analyses were used to test for significant differences between groups. A *P* value of < 0.05 was considered statistically significant.

## Results

### Demographics

By the end of July, 700 questionnaires were returned from the 69 hospitals. The response rate was 84.5% (700/828). Twenty-one questionnaires were excluded because of poor quality (with missing data or contradictory answers); thus, 679 questionnaires were included in the final analysis. A total of 190 (28.0%) pediatricians, 173 (25.5%) pharmacists, 176 (25.9%) nurses and 140 (20.6%) administrators replied. Among these, 109 (16.1%) had senior titles, 336 (49.5%) had intermediate titles and 234 (34.5%) had junior titles (Table 1).

**Table 1:** Demographics of the study participants

	Secondary hospital (*n* = 403)	Tertiary hospital (*n* = 276)
Profession, *n* (%)		
Pediatrician	110 (57.9)	80 (42.1)
Pharmacist	100 (57.8)	73 (42.2)
Nurse	107 (60.8)	69 (39.2)
Administrator	86 (61.4)	54 (38.6)
Professional title^a^, *n* (%)		
Junior title	150 (64.1)	84 (35.9)
Intermediate title	195 (58.0)	141 (42.0)
Senior title	54 (49.5)	55 (50.5)

Note. ^a^ Health workers in China are often classified as “senior”, “intermediate” or “junior” title according to their skill levels and specialization. For example, doctors with junior title refer to residents, intermediate title refer to attending physicians and senior title refer to director physicians or assistant director physicians

### Current practice and management of pediatric off-label drug use in Shanghai

Nearly half (47.9%) of the pediatricians acknowledged that they had prescribed off-label drugs. No significant differences in the rate of off-label prescribing were found among pediatricians with different professional titles or pediatricians in different level hospitals (Table 2). When prescribing an off-label medicine, only 41 (21.6%) pediatricians would always obtain consent. Most (88.4%) of the pharmacists acknowledged that they had dispensed off-label medicines. More than half of them required the prescribers to note the reasons for off-label use and a signature or consulted related literature to find evidence before dispensing off-label medicines. The majority of nurses (85.2%) stated that they would confirm with the prescribers for rationality and pay attention to ADRs when administering off-label medicines. Common reasons for off-label prescribing were as follows: lack of pediatric dosage information (55.3%), Summary of Product Characteristics (SmPC) had not been revised and updated about new indications (43.7%), SmPC was inaccurate or ambiguous (29.5%), and a lack of appropriate pediatric formulations (25.3%). The most common category of off-label prescribing in children was dosage (41.7%).

**Table 2:** Comparisons of off-label prescribing rates between pediatricians with different clinical backgrounds

	Have you ever prescribed off-label drugs		χ^2^	*P*
	Yes	No		
Professional title, *n* (%)				
Junior title	14 (35.9)	25 (64.1)	2.84	0.241
Intermediate title	44 (50.6)	43 (49.4)		
Senior title	33 (51.6)	31 (48.4)		
Practice setting, *n* (%)				
Secondary hospital	47 (42.7)	63 (57.3)	2.80	0.095
Tertiary hospital	44 (55.0)	36 (45.0)		

Less than half (42.0%) of the participating hospitals had developed internal guidance on off-label drug use. The off-label process included all or part of the following procedures: applying with relative information and evidence, being approved by the ethics committee, being approved by the pharmacy administration committee, obtaining informed consent, and monitoring the adverse reactions. However, approximately half of the respondents reported that they did not adhere to the guideline and that it had barriers to utilization. Tertiary hospitals were more likely than secondary hospitals to develop internal protocols for off-label drug use (59.3% vs 30.9%, χ^2^ = 5.4, *P* = 0.02).

### Knowledge and awareness of pediatric off-label drug use and the *Chinese Expert Consensus of Pediatric Off-Label Drug Use*

**Fig. 1:**
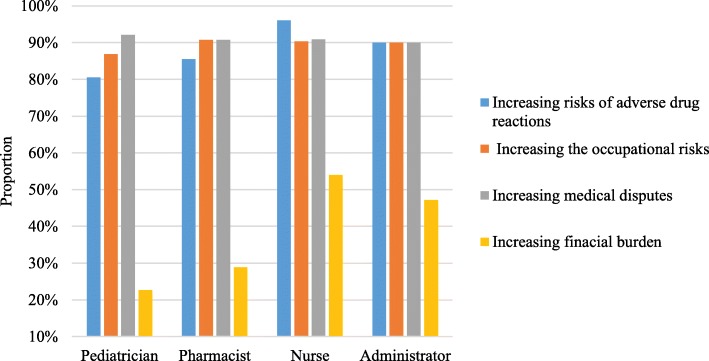
Healthcare professionals’ risk concerns regarding off-label drug use

Five hundred and seventy-four respondents (84.5%) declared they were familiar with the term “off-label drug use”, and pediatricians were more familiar with the concept than other healthcare professionals were (Table 3). Four hundred and thirty-nine (64.7%) of the respondents believed that it was not illegal to use off-label drugs. However, the vast majority of the respondents realized that there were potential risks to the use of off-label drugs such as increasing the risks of ADRs, increasing the occupational risks of healthcare professionals and increasing medical disputes. Nurses were the most concerned about ADRs when compared with the other groups of healthcare professionals (Fig. 1). More than half (70.1%) of the respondents thought off-label drug use was appropriate when it was in the best interest of the patient on the basis of credible, published scientific data support. The majority of respondents (83.5%) agreed that parents or guardians should be informed when an off-label medicine was prescribed for their children, and the level of recognition was significantly correlated with professional titles (senior vs intermediate vs junior: 73.3% vs 83.9% vs 87.6%, χ^2^ = 10.99, *P* = 0.004). Almost all (92.9%) of the respondents expressed the desire for more education and guidance on off-label drug use.

**Table 3:** Comparisons of the off-label familiarity between professionals with different clinical backgrounds

	Are you familiar with the definition of off-label drug use		χ^2^	*P*
	Yes	No		
Profession, *n* (%)				
Pediatrician	179 (94.2)	11 (5.8)	24.87	< 0.001
Pharmacist	154 (89.0)	19 (11.0)		
Nurse	136 (77.2)	40 (22.8)		
Administrator	124 (88.6)	16 (11.4)		
Professional title, *n* (%)				
Junior title	192 (82.1)	42 (17.9)	9.01	0.011
Intermediate title	303 (90.2)	33 (9.8)		
Senior title	98 (89.9)	11 (10.1)		
Practice setting, *n* (%)				
Secondary hospital	348 (86.4)	55 (13.6)	0.86	0.353
Tertiary hospital	245 (88.8)	31 (11.2)

More than half (54.3%) of the respondents did not know about the *Chinese Expert Consensus of Pediatric Off-Label Drug Use*. Pharmacists had the highest awareness rate (59.5%), followed by pediatricians (47.9%), administrators (41.4%) and nurses (32.9%). Significant differences were found between different professional groups (χ^2^ = 26.27, *P* = 0.000). Compared to respondents with lower professional titles, respondents with higher professional titles were more likely to be aware of the consensus. There were no differences in awareness by hospital characteristics (Table 4).

**Table 4:** Comparisons of awareness rates of the expert consensus between different groups

	Awareness rate	χ^2^	*P*
Profession, *n* (%)			
Pediatrician	91 (47.9)	26.27	< 0.001
Pharmacist	103 (59.5)		
Nurse	58 (32.9)		
Administrator	58 (41.4)		
Professional title, *n* (%)			
Junior title	105 (44.9)	9.40	0.009
Intermediate title	141 (42.0)		
Senior title	64 (58.7)		
Practice setting, *n* (%)			
Secondary hospital	180 (44.7)	0.39	0.531
Tertiary hospital	130 (47.1)		

### Barriers to implementation of the Chinese expert consensus of pediatric off-label drug use

More than half (55.4%) of the respondents declared they did not to adhere to the process proposed in the consensus and that barriers existed in its utilization, including a lack of time to provide information sources and evidence of off-label use (69.8%), no available expert group for off-label drug use (52.6%), no monitoring system for ADRs (48.3%), no database of the off-label drugs (46.8%), and no ethics committee or pharmacy administration committee (14.7%). Nearly half (45.4%) of the respondents considered it difficult to obtain written consent from parents or guardians. When asked if it was necessary to implement the process in different level hospitals, almost half (49.2%) of the respondents believed that the process may not be generalizable to all situations as limited by skill and equipment. More than 80% of the respondents believed that it was appropriate to use grading management regarding off-label drug use and to constrain the right to prescribe off-label medicines. Nearly all (93.8%) of the respondents called for a special department to track research into off-label drug use and to establish a national formulary of pediatric off -label drugs.

## Discussion

In China, and throughout the world, pediatricians are increasingly put under ethical and professional obligations to ensure safe drug therapy. Therefore, a thorough understanding of the issues surrounding off-label prescribing is essential. The present study was conducted to evaluate the awareness of off-label drug use among medical staff involved in pediatric off-label drug use and to assess barriers to the implementation of expert consensus in Shanghai, China. To the best of our knowledge, this is the first study that contemporaneously assessed a range of Chinese healthcare professionals in terms of their views on off-label prescribing in children and the current management situation.

Overall, some of the findings in this survey were consistent with the results of earlier studies carried out in Western countries such as that off-label drug use was widespread and common, that the majority of healthcare professionals were familiar with the concept of off-label drug use, and that a lack of pediatric dosage information was one of the main reasons for off-label prescribing, while dosage was highlighted as the most common category of off-label drug use [20, 21]. With regard to informed consent, the rate reported for such practices remained low. Only 31% of hospital based pediatricians admitted to obtaining informed consent when they were prescribing an off-label medicine in Scotland [22], with a similar rate reported in a Northern Ireland study (30.7%) [16]; In this study, we found a lower rate of 21.6%, despite 83.5% of the respondents agreeing that parents or guardians should be informed when an off-label medicine was prescribed for their children. Almost half of the respondents felt that it was difficult to obtain written consent from parents or guardians which may be attributable to the health workers’ heavy workload and the serious status of the doctor-patient relationship in China. An interesting result was that a lower percentage of higher level healthcare professionals who had more clinical expertise and experience agreed with obtaining informed consent. This finding may be attributed to their high confidence in their medical practice. These results emphasize the need to improve communications between parents and healthcare professionals especially those with higher level titles in situations when off-label prescriptions are being issued. In Australia, different processes for informed consent have been proposed according to different levels of evidences in off-label prescribing [23]. We may learn lessons from these processes when making regulations.

Off-label prescribing is legal in the United States and European Union countries [4]. In India, amendments to the Indian Medical Council Act made off-label prescribing illegal because of the ignorance of patients and the domination of pharmaceutical companies in the prescribing patterns in India [24]. However, no legal regulations in regard to off-label prescribing in China have been identified; thus, a complex ethical and legal situation might develop, particularly regarding the question of medical liability. In this study, nearly two-thirds of the respondents considered it legal to use off-label drugs and believed that it permits innovation in clinical practice, particularly when approved treatments have failed. At the same time, the respondents were concerned about the related risks for both patients and themselves. Among them, nurses paid more attention to ADRs than the other professionals did. A discrepancy was found when these results were compared with a study conducted in Northern Ireland, which showed that pediatric nurses were the least concerned about safety issues among various groups of healthcare professionals [16]. This finding may reflect that nurses in Shanghai may be better educated regarding ADRs.

There is no clear definition regarding the right to prescribe off-label drugs worldwide. In the UK, doctors, dentists, independent nurses and pharmacist prescribers are allowed to prescribe off-label medicines by the Medicines and Healthcare Products Regulatory Agency [25]. In China, only doctors have prescription rights. In this survey, more than half of the respondents suggested the need to constrain the right to prescribe off-label drugs to prevent the abuse of off-label drugs.

In view of there being no previous studies assessing barriers to guideline implementation focused on off-label drug use, another theme of this study was to evaluate the barriers to clinical practice of the existing guideline in Shanghai, China. To promote awareness of off-label use among pediatric healthcare professionals and to provide a practical approach to off-label prescribing that would ultimately result in improved drug use in children, the *Chinese Expert Consensus of Pediatric Off-Label Drug Use* written by the Chinese Pediatric Society was published in the Chinese Journal of Pediatrics in 2016, and a process for off-label drug use was proposed. Unfortunately, less than half of the respondents were aware of the expert consensus’s existence, suggesting inadequate dissemination and uptake of the expert consensus. Therefore, a lack of awareness about the expert consensus was perceived as the first barrier preventing guideline use. Then, an important barrier was related to the consensus itself as a lack of applicability of the recommended processes in different level hospitals. Thus, a more feasible national guideline for off-label drug use tailored to the local setting is urgently needed in China. Hanbin Wu from the Tongji University School of Medicine proposed grading management to address innovative off-label medication use in China, which provided some references for medical institutions [26]. Furthermore, healthcare professionals’ inadequate training in off-label use was speculated to be another barrier to guideline use since almost all respondents expressed the desire for more education and guidance on off-label drug use. Overall, these results not only identified some barriers to the current expert consensus implementation but also emphasized the need for training on this topic. This information may be useful for healthcare policymakers, regulatory bodies and other stakeholders involved in regulatory decisions for off-label drug use.

### Study limitations

The nature of this survey meant that we were only able to superficially explore this area and more detailed work would be of value in the future, including exploring off-label practice nationwide and strategies to overcome barriers. Self-report questionnaires have limitations with regard to accurately assessing prescribing, dispensing and medicine administration practice. The outcomes may be inevitably influenced by individual subjective factors. However, it is hoped that the anonymity of the questionnaire encouraged honesty.

## Conclusions

Pediatric off-label drug use is a common practice in Shanghai, China. Chinese healthcare professionals have a recognition of the concept but a low awareness rate of the *Chinese Expert Consensus of Pediatric Off-Label Drug Use.* At the same time, a range of barriers to the expert consensus implementation have been identified. Based on the results, we suggest the following: (1) Off-label drug use has its rationality and necessity, while the potential risks cannot be ignored, a legally recognized national guideline with a broad scope of application is urgently needed in China. (2) Targeted pediatric healthcare professionals’ education and training on off-label drug use should be taken into consideration to guide clinical practice and improve guideline adherence. As a pilot study in China, our results should be of interest to off-label stakeholders in other cities and a subsequent nationwide survey may be conducted in the future.

## Additional file

Additional file 1: Detailed questionaire. (DOCX 39 kb)

## Data Availability

The datasets used and/or analyzed during the current study are available from the first or corresponding author on reasonable request.

## Abbreviations

ADRs: Adverse drug reactions
SmPC: Summary of Product Characteristics
SPQCC: Shanghai Pediatric Clinical Quality Control Center
