# Supplementary material for: Current practice and awareness of pediatric off-label drug use in Shanghai, China -a questionnaire-based study
Source: BMC Pediatr. 2019 Aug 13;19:281. doi: 10.1186/s12887-019-1664-7 (PMC6691537; doi:10.1186/s12887-019-1664-7)
Supplement: Supplementary file 1 — Detailed questionaire. (DOCX 39 kb) [file 12887_2019_1664_MOESM1_ESM.docx]

**Pediatric off-label drug use in Shanghai China**

**Dear health professionals:**

Off-label drug use is the administration of a drug for conditions outside the product license with respect to the dose, age, route of administration, indications or contra-indications etc. Due to the lack of drugs specifically designed and marketed for children, off-label drug use is very common in pediatric drug treatment. Although the existence of off-label drug use has its rationality, it may cause several problems. In some developed countries, national legislations, regulations or guidelines concerning off-label drug use have been established and rational off-label drug use is allowed. However, there is no clear description according to Chinese laws. In 2016, the Chinese Expert Consensus of Pediatric Off-Label Drug Use was published in the Chinese Journal of Pediatrics, which was written by the Chinese Pediatric Society. The objective of the survey is to assess current practice and awareness of pediatric off-label drug use as well as barriers to the implementation of the current expert consensus in Shanghai.

Would you please spare a few minutes to complete the questionnaire? Your answers will be kept confidential, thank you for your cooperation!

**Part I General information**

1. **The** **name of your hospital**
2. **The level of your hospital**

A Primary hospital B Secondary hospital C Tertiary hospital

1. **The character of your hospital**

A Specialized children's hospital

B General hospital

C Others

1. **Which department you are working in**
2. **What is your profession**

A Doctor

B Pharmacist

C Nurse

D Administrator

1. **What is your professional title**

A Junior

B Intermediate

C Senior

**Part II Current practice and management of pediatric off-label use**

1. **Have you ever prescribed off-label drugs** (answered by doctor)

A Yes B No

1. **Why did you** **prescribe off-label drugs** (answered by doctor)

A Lack of pediatric dosage information

B Drug instructions are inaccurate or ambiguous

C Summary of Product Characteristics was not revised and updated about new indications.

D Lack of appropriate pediatric formulations.

E Required by the family members

F Unaware of off-label prescribing

G Misled by pharmaceutical enterprises

H I have never prescribed off-label drugs

others:

1. **Do you obtain informed consent from parents or guardians when prescribing off-label medicines** (answered by doctor)

A Always

B Sometimes

C Never

1. **Have you ever dispensed off-label medicines** (answered by pharmacist)

A yes

B No

1. **When you dispense off-label medicines, what do you do** (answered by pharmacist)

A Ask superior for instructions and make a note

B Confirm with the doctor and dispense medicines

C Consult the related literature to find evidence, then dispense medicines

D Refuse to dispense

Others

1. **When you execute off-label orders, what do you do** (answered by nurse)

A Confirm with the doctor, acquire off-label reason and evidence, make a note and observe adverse drug reactions

B Perform the doctor's order as long as he/she signature

C Refuse to perform

Others

1. **What is the most common category of off-label prescribing in children**

A Age

B Indication

C Dosage

D Route of administration

Others

1. **Any guideline or regulations of off label use in your hospital?**

A yes

B No

1. **What is the process of off-label drug use in your hospital**

A Applying with relative information and evidence

B Being approved by the ethics committee

C Being approved by the pharmacy administration committee

D Obtaining informed consent

E Monitoring the adverse reaction

1. **Do you think the off-label drug use process in your institution is practical for implementation**

A Yes

B No, it has barriers

1. **Do you adhere to your internal guideline when come across off-label drug use**

A. Yes

B. No

**Part III** **Knowledge and awareness of pediatric off-label drug use and the *Chinese Expert Consensus of Pediatric Off-Label Drug Use***

1. **Are you familiar with the definition of off-label drug use**

A Yes B No

1. **Do you think off-label prescribing is illegal**

A Yes, it is

B No, it isn’t

1. **What are the risks of off-label drug use**

A Increase the risk of adverse reactions

B Increase medical disputes

C Increase occupational risks of healthcare professionals

D Increase the patient's financial burden

F Other

1. **When off-label use is appropriate in your mind**

A If there is a medical need impacting the quality of life or threatening life, and no approved/labelled medicine is available

B As part of an approved medical research project

C There is high level evidence to support the usage

D Obtaining informed consent

E Not acceptable in any cases

F Other

1. **What are the barriers for labelling for children**

A It costs too much

B It takes too much time

C Pharmaceutical companies lack the motivation to update the labels

D It is difficult to carry out clinical trials in pediatric population

E Other

1. **What role can pharmacists play in updating drug instructions**

A Participate in the management of clinical trials

B Feedback the latest usage of a medicine to the pharmaceutical company

C Evaluate the safety and efficacy of the marketed drug and feedback to the pharmaceutical company

Other

1. **What can be the information source of off-label prescribing in your opinion**

A Clinical guidelines from both home and abroad

B Drug instructions abroad

C Literatures (including system evaluation and meta)

D Expert consensus from both home and abroad

E The national formulary

F The hospital formulary

G Clinical experience

1. **In 2016, the *Chinese Expert Consensus of Pediatric Off-Label Drug Use* was published in the Chinese Journal of Pediatrics, which was written by the Chinese Pediatric Society** [**http://www.cmaped.org.cn/CN112140201602/864534.htm**](http://www.cmaped.org.cn/CN112140201602/864534.htm)**. Do you know it**

A Yes

B No

1. **Process for off-label drug use was recommended in the consensus. Including: (1) applying with relative information and evidence of off-label drug use; (2) being evaluated by the expert group of off-label drug use; (3) being approved by the ethics committee and/or the pharmacy administration committee; (4) obtaining informed consent; (5) monitoring the adverse drug reactions; (6) establishing a database of the off-label use drugs and updating regularly. Do you know the process**

A Yes

B No

1. **What is the obstacle of the “Expert Consensus” implemented in your hospital**

A It was time-consuming to provide information source and evidence

B There is no expert group of off-label drug use in my hospital

C There is no ethics committee in my hospital

D There is no pharmacy administration in my hospital

E It is difficult to obtain written consent from parents or guardians

F There is no drug adverse reaction monitoring system in my hospital

G There is no database of the off-label use drugs in my hospital

Other

1. **If it is necessary to implement the process in different level hospitals**

A Yes, it is beneficial to the standardized management of off-label use

B No, it is challenging for primary hospitals and secondary hospitals as limited by skill and equipment

Other

1. **If all off-label medicines should to go through the process in your opinion**

A Yes

B No

1. **What kind of off-label medicines need not go through the application procedure**

A No exceptions

B As a standard treatment recognized by the department of health administration

C With sufficient evidence and included in the list of the hospital’s formulary

D Repeated off-label use that have been approved before

Other

1. **Is it acceptable to prescribe an off-label medicine** **in emergency without application**

A Yes, it is

B No, it is’t

1. **What materials should be provided when applying for off-label drug use**

A Drug information, including generic name, trade name, manufacturers, dosage form, specifications

B Summary of Product Characteristics

C The objective and the therapeutic regimen of off-label drug use

D The evidences with level

E The potential risk and the emergency plan

F Informed consent

Other

1. **What kind of off-label drug use must be approved by Ethics Committee in your opinion**

A All off-label drug use must be approved by Ethics Committee

B Drugs with serious side effect

C The drug undergoing clinical trial

Other

1. **Who should be included in the expert group of off-label drug use**

A Pharmacist

B Doctor

C Administrator

D Nurse

Other

1. **Should parents or guardians be told when an off-label medicine is prescribed for their children**

A Yes

B No

C It depends

1. **Do you think grading management is fit for the management of off-label drug use in China**

A yes

B No

1. **What should be taken into consideration during grading management of off-label drug use**

A The level of evidence

B Safety

C Efficacy

D Economic efficiency

E The right to prescribe off-label medicine

F Target population

G History of medical disputes

1. **In order to prevent the abuse of off-label drugs, is it appropriate to constrain the prescription right of off-label drug use**

A No, all doctors with medical license have the right

B Yes, doctors with medical license who have been trained on off-label prescribing have the right

C Yes, the hospital level and the doctor’s professional title should be taken into consideration

1. **What is the role of the pharmacists in off-label drug use**

A Review the off-label prescription and feedback to doctors

B Track research into off-label drug use and to establish a formulary of off-label medicines

C Analyze the off-label prescriptions regularly and feedback to doctors

D Participate in the application management

1. **What should be checked when dispensing off-label drugs**

A Approved document

B Off-label prescription

C Informed Consent Form

Other

1. **Is it necessary for pharmacists to intervene when off-label drug use happen**

A Yes, it is

B Dispensable

C No, it intervenes the normal routine

1. **What details should be paid attention to when you are prescribing an off-label medicine**

A The reason and the therapeutic regimen for off-label drug use must be detailed in the records

B Informed consent

C Monitoring of the adverse effects of drugs

D Assessing the efficiency periodically

E Other

1. **There are unpredictable risks for patients during off-label drug use, which monitoring system is suitable for your daily clinical work**

A The prescribers are responsible for monitoring the adverse drug reactions

B Monitored by the existing adverse reaction monitoring system

C Setting up a special department for monitoring the adverse drug reactions of off-label use

D Other

1. **What information should be collected by the off-label adverse reaction monitoring system**

A General information and the diagnosis of patients

B Drug information, including generic name, trade name, manufacturers, dosage form, specifications

C Category of off-label drug use

D Therapy in detail

E Adverse reaction in detail

F Solution

G Other

1. **Who should take the responsibility for the adverse reaction when patients have signed the informed consent**

A If the adverse reactions have been written in drug labels, doctors should not take responsibility

B If doctors ignored that the patient has the specific contraindication for this drug, doctors would take the responsibility

C The doctors take the full responsibility under any circumstances

D The doctors, pharmacists and hospitals take the responsibility together

E Other

1. **Do you get adequate knowledge of off-label drug use in children**

A Yes

B No

1. **Is it necessary to provide education and training on off-label drug use to medical staffs**

A Yes, it is

B No, it is not necessary

1. **Is it necessary to set up a special national department to track research into off-label drug use and to establish a national formulary of pediatric off -label use drugs and update it regularly**

A Yes，it is necessary

B No, medical institutions can set up one by themselves

1. **Do you think the costs of off-label drug use should be covered by insurance**

A No reimbursement for fees under any circumstances

B Reimburse patients who are in serious illness with evidence support the treatment

C Reimburse patients if the off-label medicines are recorded in national formulary

1. **How can we improve the awareness rate of the "Expert Consensus of Pediatric Off-Label Drug Use”**

A The authority needs to pay more emphasis on its dissemination to promote it widely

B Strengthening the training of medical professional on off-label drug use in medical institutions

C Strengthening education on off-label drug use by academic organizations

D Other

**Thank you very much indeed for assisting with this research!**
